# Field releases of the exotic parasitoid *Trissolcus japonicus* (Hymenoptera: Scelionidae) and survey of native parasitoids attacking *Halyomorpha halys* (Hemiptera: Pentatomidae) in Michigan

**DOI:** 10.1093/ee/nvad102

**Published:** 2023-10-06

**Authors:** Olivia Simaz, Julie Michaelson, Julianna K Wilson, Elijah Talamas, Larry Gut, John Pote, Marianna Szűcs

**Affiliations:** Department of Entomology, Michigan State University, East Lansing, MI, USA; Department of Entomology, Michigan State University, East Lansing, MI, USA; Department of Entomology, Michigan State University, East Lansing, MI, USA; Florida Department of Agriculture and Consumer Services, Bureau of Entomology, Nematology and Plant Pathology, Division of Plant Industry, Gainesville, FL, USA; Department of Entomology, Michigan State University, East Lansing, MI, USA; Department of Entomology, Michigan State University, East Lansing, MI, USA; Department of Entomology, Michigan State University, East Lansing, MI, USA

**Keywords:** brown marmorated stink bug, release size, release frequency, biological control, adventive parasitoid

## Abstract

An adventive population of the exotic parasitoid wasp, *Trissolcus japonicus* (Ashmead) (Hymenoptera: Scelionidae), discovered in Michigan in 2018, is a promising biological control agent of the invasive *Halyomorpha halys* (Stål) (Hemiptera: Pentatomidae). Following its discovery, field releases of *Tr. japonicus* were conducted over 2 yr in southern Michigan, to test how release size or release frequency impacts establishment. Sentinel eggs of *H. halys* and of the native *Podisus maculiventris* (Say) (Hemiptera: Pentatomidae) were used alongside yellow sticky cards to monitor parasitoids. In 2019 and 2020, 7,200 *Tr. japonicus* were released at 16 sites. Monitoring between 2019 and 2021 yielded only 49 individuals. The captures suggest reproductive activity and overwintering success in the field but do not allow for evaluation of best release methods. Parasitism by native parasitoids was below 7%, which is similar to other states and unlikely to provide sufficient control of *H. halys*. The placement of sentinel eggs or sticky traps either in the lower or middle canopy of trees did not influence parasitoid capture rates. Frozen and fresh *H. halys* sentinel eggs were attacked at the same rate, but more native parasitoids emerged from frozen eggs. We did not find signs of nontarget effects on *P. maculiventris* thus parasitism rates overall were very low. These results could indicate dispersal of *Tr. japonicus* from the release sites or slow population growth. The latter may be due to the relatively low densities of *H. halys* in Michigan or may stem from the small founding size of our laboratory colony.

## Introduction

Invasive species are sometimes accompanied by natural enemies from their native ranges (Kaser and Heimpel 2015, Wheeler et al. 2017, Stahl et al. 2019, Hogg et al. 2021, Abram et al. 2022). Such co-evolved natural enemies are often used for biological control of invasive species (Heimpel and Mills 2017, Van Driesche et al. 2020, Mason 2021). However, the natural dispersal and population growth of adventive natural enemies can be slow in areas that are distant from the original point of introduction. Human-assisted redistribution can help to increase the geographic range and population densities of these adventive natural enemies, potentially accelerating biological control. In addition, new releases of a species across the landscape where they are either absent or may be present at very low densities could be used to assess basic mechanisms that mediate establishment success of species in novel environments.

The brown marmorated stink bug, *Halyomorpha halys* (Stål) (Hemiptera: Pentatomidae) is an invasive insect from eastern Asia that was introduced to North America, Europe, and South America (Haye et al. 2015, Faúndez and Rider 2017, Leskey and Nielsen 2018). It is highly polyphagous and has become a primary agricultural pest of multiple fruit and vegetable crops (Leskey and Nielsen 2018). Since its introduction to the United States in 1996, *H. halys* has spread to at least 47 states and 4 Canadian provinces (Hoebeke and Carter 2003, Northeastern IPM Center 2022). A suite of native parasitoids from 4 genera, *Anastatus* Motchoulsky (Eupelmidae), *Ooencyrtus* Ashmead (Encyrtidae), *Telenomus* Haliday, and *Trissolcus* Ashmead (Scelionidae) were found to attack *H. halys* eggs in North America, but they are ineffective at controlling the pest, and their parasitism rates have remained low (<10%) in the United States for the past 2 decades (Abram et al. 2017).

In 2014, adventive populations of an exotic egg parasitoid, *Trissolcus japonicus* (Ashmead) (Hymenoptera: Scelionidae) were found first in Maryland and in subsequent years in multiple mid-Atlantic and western states (Talamas et al. 2015, Herlihy et al. 2016, Hedstrom et al. 2017, Milnes and Beers 2019). *Trissolcus japonicus* is an oligophagous parasitoid that can parasitize 50–80% of *H. halys* eggs in its native range and is considered the most promising biological control agent against *H. halys* (Yang et al. 2009, Zhang et al. 2017). It has been tested in quarantine in the United States since 2007 as the primary candidate for a classical biological control program (Talamas et al. 2015). Host-specificity tests showed that it can develop in at least 12 native stink bug species in North America (Hedstrom et al. 2017, Botch and Delfosse 2018, Milnes and Beers 2019). Nevertheless, once it fortuitously showed up, multiple states started conducting augmentative releases to increase its geographic distribution and population densities (Jentsch 2017, Lowenstein et al. 2019, Milnes and Beers 2019).

*Halyomorpha halys* was first detected in Michigan in 2010 with *Tr. japonicus* detected 8 yr later (Jarrett et al. 2019). The parasitoid likely arrived from neighboring Ohio, where it was discovered just a year prior (Northeastern IPM Center 2022). Despite extensive sampling across Michigan that involved placing 189 *H. halys* sentinel egg masses over 10 field sites, *Tr. japonicus* was captured only at a single location where 3 females and 2 males emerged from 1 *H. halys* egg mass (Jarrett et al. 2019). A colony of *Tr. japonicus* was built from this initial capture and releases were conducted in 2019 and 2020. These releases may be considered classical biological control introductions given that an exotic species was redistributed to new locations.

In 2019, the releases aimed to test the importance of release size and in 2020 the release frequency for successful establishment. We intended to test these mechanisms because little is known how to release biological control agents to ensure their establishment. Less than 33% of predator and parasitoid introductions against insect pests have led to establishment worldwide (Cock et al. 2016), and in North America, only around 54% of the released parasitoids have become established since 1985 (van Driesche et al. 2020). Multiple mechanisms may influence establishment, including abiotic conditions, species-specific biological traits, stochastic processes, population density effects, or the level of genetic variation within the released population (Fauvergue and Hopper 2009). However, the most consistent predictor of establishment success across a wide range of species is found to be propagule pressure: the combined size and frequency of introductions (Lockwood et al. 2005, Colautti et al. 2006, Blackburn et al. 2015). Hence, we planned to test these factors with our augmentative releases.

We used *H. halys* sentinel eggs and yellow sticky traps to monitor *Tr. japonicus* establishment success, the diversity of native parasitoids, and their parasitism rates on *H. halys* between 2019 and 2021. With our monitoring efforts we also tested how the use of fresh or frozen *H. halys* sentinel eggs may influence parasitism. The freezing process is hypothesized to increase parasitism by native species because it kills the developing stink bug embryo, thereby removing any potential immune response or defense mechanism that could counteract parasitism (Herlihy et al. 2016). However, evidence for this has been mixed with some studies finding increased parasitism in frozen eggs while others finding no difference in parasitism between fresh and frozen eggs (reviewed in Abram et al. 2017, McIntosh et al. 2019). We also tested how the placement of sentinel eggs and yellow sticky cards either lower or higher in the tree canopy may impact parasitism. There is some evidence that *Tr. japonicus* may prefer the middle and upper canopy of their native host, tree-of-heaven [*Ailanthus altissima* (Mill.) Swingle], while native parasitoids were equally likely to be captured in the low, mid, or upper canopies of this exotic tree species (Quinn et al. 2019). We expanded on these findings by evaluating how parasitoid capture rates are influenced by sampling height on a wide diversity of tree species that are common in Michigan. Finally, given that *Tr. japonicus* can attack multiple native stink bug species in the laboratory, but little is known of any potential nontarget attack in the field (Gariepy and Talamas 2019, Milnes and Beers 2019), we also tested whether eggs of a native predatory stink bug species, *Podisus maculiventris* (Say) (Hemiptera: Pentatomidae) may be attacked by the released parasitoids.

## Materials and Methods

### Insect Rearing

The Michigan colony of *Tr. japonicus* originated from 5 individuals, 2 males and 3 females that emerged from a single *H. halys* sentinel egg mass placed at the Student Organic Farm on the Michigan State University (MSU) campus (42.6749, −84.4897) in August 2018 (Jarrett et al. 2019). Surveys conducted in 2018 and prior years across Michigan yielded this single capture event of *Tr. japonicus* (Jarrett et al. 2019). Augmentation of our colony from out-of-state individuals was not possible given the restrictions on intentional interstate movement of this adventive parasitoid. Wasps were reared by providing either fresh (<72 h old) or frozen (at −80 °C) *H. halys* egg masses to groups of 5–15 mixed-sex individuals for 3–7 days for oviposition in 10-dram plastic vials. A drop of honey was placed on the lids to provision wasps. Vials were kept in an incubator at 20 °C and 70% humidity with a photoperiod of 16:8 L:D. Adult wasps emerged 14–21 days following oviposition.

*Halyomorpha hal*y*s* eggs from colonies maintained on MSU campus or from the New Jersey Department of Agriculture Phillip Alampi Beneficial Insect Laboratory rearing facility were used for rearing *Tr. japonicus*. Adult *H. halys* from MSU campus colonies were reared in mesh cages (30 × 30 × 60 cm) by keeping 40–60 mixed-sex adults in each cage at 25 °C, 50–75% humidity, and 16:8 L:D photoperiod in a climate-controlled room. Nymphs were housed in 236 ml clear, square plastic containers. A 6 cm^2^ hole was cut on the lids and covered with gauze to provide ventilation. All stages were provided water via dental wicks inserted into 60 ml cups filled with water. Their diet included organic green beans, snap peas, broccoli, carrots, apples, and mixed nuts.

The native stink bug *P. maculiventris*, used to assess nontarget effects of *Tr. japonicus*, was reared in groups of 40–50 in 1.2 liter clear, round plastic containers at 25 °C, 50–75% humidity, and 16:8 L:D photoperiod in a climate chamber. Water was provided via dental wicks inserted in 60 ml cups filled with water. Their diet included wax worms (Top Hat Cricket Farm Inc., Portage, Michigan) and organic green beans. For both stink bug colonies, eggs were collected 2–3 times weekly from the green bean leaves and paper towels and used either to rear parasitoids, as sentinel eggs in the field, returned to the colony, or were frozen at −80 °C for later use.

### Site Selection and Experimental Design

Field releases were conducted in 2019 and 2020 to test how different release methods may impact establishment success of *Tr. japonicus*. We used a network of growers who have had a history of collaboration with researchers from MSU and whom we could rely on to provide site access for us during the season while excluding the public to select 24 field sites. Site selection criteria included minimal disturbance of the release and monitoring locations, and up-to-date spraying information so we could time the releases and monitoring in spray-free periods. A subsequent criterion for choosing field sites was that they have a history of relatively high densities of *H. halys* in the region as assessed by a multiyear monitoring effort prior to this study (Wilson, *unpublished data*). Another criterion was to have at least one side of the field bordered with a woodlot that can provide habitat for *H. halys* and *Tr. japonicus* and where pesticides are not sprayed. These woodlots contained a diversity of coniferous and deciduous species including sugar maple (*Acer saccharum*), red maple (*A. rubrum*), American beech (*Fagus grandifolia*), white pine (*Pinus strobus*), eastern cottonwood (*Populus deltoides*), red oak (*Quercus rubra*), white oak (*Q. alba*), black locust (*Robinia pseudoacacia*), dogwood (*Cornus* sp.), viburnum (*Viburnum* sp.), and mulberry (*Morus* sp.). Given the above criteria, the chosen study sites were mostly apple orchards (*n* = 16) managed either conventionally (*n* = 14) or using low input or organic methods (*n* = 2). Eight sites had a mix of crops of both different fruits and vegetables and were either managed conventionally (*n* = 1) or with low input or organic means (*n* = 7) (Supplementary Table S1).

**Fig. 1.**
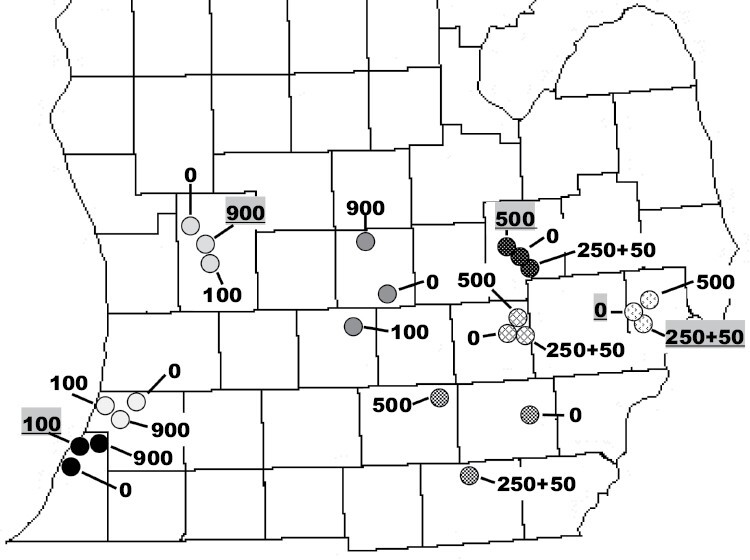
Study sites for *Tr. japonicus* releases in 2019 (solid fill) and in 2020 (pattern fill) in lower Michigan. Different shaded circles indicate the 4 regional blocks each year within which sites were assigned to different *Tr. japonicus* release treatments. The 5 underlined release numbers indicate the sites where *Tr. japonicus* was recaptured in 2020 and 2021. For exact site coordinates see Supplementary Table S2.

In 2019, twelve study sites were identified in southwestern and central Michigan with the aim to assess the importance of release size for establishment success (Fig. 1). Given the mostly western location of these sites and for ease of reference these sites will be referred to as “western”. A randomized complete block design was used with 3 release treatments (0, 100, or 900 individuals) assigned randomly within each block, replicated over 4 blocks. The blocks represented clusters of sites that were in geographic proximity, but that were at least 4 km apart (Fig. 1). These releases were monitored in 2019 using a mix of fresh (<72 h old) and frozen *H. halys* sentinel eggs, in 2021 using only frozen eggs (Table 1), and with yellow sticky traps (Trécé AM no-Bait traps) in 2020 and 2021. *Podisus maculiventris* eggs were deployed to assess possible nontarget attacks only in 2019. The relative density of *H. halys* was measured using 4 pyramid traps at each of the 12 study sites. Pyramid traps were placed 7 m apart parallel to the tree line 10 m within the woodlots bordering the orchards.

**Table 1.** Results of monitoring efforts following *Tr. japonicus* releases at 24 sites in Michigan. In 2020, 7 individuals of *Tr. japonicus* emerged from 1 egg *H. halys* egg mass. In 2022, 39 *Tr. japonicus* individuals emerged from 6 *H. halys* egg masses. Parasitism rates for all other egg masses refer to native parasitoids

*Trissolcus japonicus* release	2019	2019	2020	2019/2020
Sites monitored	12 western	4 western	12 eastern	24 east/west
Monitoring dates	June–Sep, 2019	June–Sep, 2019	June–Sep, 2020	July 2021
Sentinel eggs used	*H. halys*	*P. maculiventris*	*H. halys*	*H. halys*
Sentinel eggs deployed	840	285	430	240
Sentinel eggs retrieved	727	133	321	219
Sentinel eggs placed lower (1.5 m)	371 (51%)	NA	168 (52%)	219 (100%)
Sentinel eggs placed higher (3.5 m)	356 (49%)	133 (100%)	153 (48%)	NA
Frozen sentinel eggs	532 (73%)	39 (30%)	321 (100%)	219 (100%)
Fresh sentinel eggs	195 (27%)	133 (70%)	NA	NA
Egg masses parasitized	30 (4.12%)	3 (2.26%)	6 (1.87%)	30 (13.7%)
Sentinel eggs placed lower parasitized	17 (57%)	NA	2 (33%)	30 (100%)
Sentinel eggs placed higher parasitized	13 (43%)	3 (100%)	4 (67%)	NA
Frozen egg masses parasitized	17 (57%)	2 (67%)	6 (100%)	30 (100%)
Fresh egg masses parasitized	13 (43%)	1 (33%)	NA	NA
Number of emerging parasitoids	241	12	39	394
Parasitoids emerging from frozen eggs	171 (71%)	10 (83%)	39	394 (100%)
Parasitoids emerging from fresh eggs	70 (29%)	2 (17%)	NA	NA
Parasitism rate on egg level	1.18%	0.60%	0.43%	6.43%

In 2020, 12 new study sites were identified in southeastern Michigan with the aim to test how release frequency may impact establishment success (Supplementary Table S1 and Fig. 1). These will be referred to as “eastern” sites or releases throughout the text. A randomized complete block design was used with 3 release treatments: 0 (control), 500 wasps released at 1 date, or 250 wasps at 1 date and 50 more wasps 4 wk later. According to the original design we intended to release 250 wasps at the second date for the latter treatment but too few *Tr. japonicus* emerged. Hence the unbalanced release size of 500 versus 300. As described for the previous year, release treatments were assigned randomly to clusters of 3 sites, replicated in 4 blocks (Fig. 1). Frozen *H. halys* sentinel eggs and yellow sticky traps were used for monitoring in 2020 and in 2021. Four pyramid traps, placed as described above, were used to monitor *H. halys* densities at each study site.

### *Trissolcus japonicus* Releases

Adult *Tr. japonicus* wasps were released in both years within the woodlots, at least 10 m from the field edges in between the 2 middle pyramid traps that were used for *H. halys* monitoring. The parasitoids were placed in 500 ml plastic deli cups that were raised into the canopy of the closest tree centered between the pyramid traps using a 3 m long pole. Parasitoids were allowed to mate prior to releases for at least 48 h. Throughout the rearing process the sex ratio of our *Tr. japonicus* colony was female biased, with 86–88% females (Linder et al. 2023). Releases were aimed to coincide with peak *H. halys* oviposition that is estimated to occur between 460 and 734 growing degree days (base 14.17 °C starting with 1 January), based on estimates modeled for Geneva, New York (Nielsen et al. 2016). These degree days corresponded to 9–27 June in 2019 and 9–25 June in 2020 in East Lansing, Michigan. Single releases took place on June 22 or at 521 degree days in 2019 (enviroweather.msu.edu). For the 2020 field season the first releases (*n* = 250 or 500) took place on July 1 or at 873 degree days and the second releases (50 individuals added to the 250 prior release) took place on Aug 4 or at 1,674 degree days.

### Monitoring for Parasitoids

In 2019 to monitor the western releases, ten *H. halys* sentinel egg masses were deployed at each site biweekly between 24 May and 9 September for a total of 840. The ten egg masses were placed in groups of 2, half-way (3.5 m) between the 4 pyramid traps and about 3.5 m from either side of the edge pyramid traps. For each group of 2 sentinel egg masses, 1 of the egg masses was glued (Elmer’s extra strength nontoxic) to the undersides of leaves that were at the required distance at 1.5 m height. The second egg mass in each group was raised to 3.5 m height using whichever tree was half-way between the pyramid traps, by attaching a leaf from the given tree to a bamboo pole and gluing the egg mass to it. Eggs were left in the field for 48 h then brought back to the lab and incubated at 20 °C, 50–75% humidity, and 16:8 L:D photoperiod in a climate chamber. Eggs were monitored for parasitoid emergence for 2 months. At the 12 eastern sites where wasp releases took place in 2020, monitoring proceeded in a similar manner as described for the western sites by placing ten *H. halys* sentinel egg masses at each site once every 2 wk (biweekly) between 2 July and 15 September for a total of 430. In 2021, reduced monitoring was conducted by placing ten frozen *H. halys* sentinel eggs at each of the western and eastern sites just once during the season. These egg masses were all placed as described above at 1.5 m height on 5–6 July 2021 at the western, and on 19–20 July 2021 at the eastern release sites.

Potential nontarget effects of *Tr. japonicus* were monitored in 2019 by placing at least 5 fresh *P. maculiventris* egg masses, depending on availability, at 3.5 m height biweekly at western sites that received 900 wasps where the potential for attack was highest. *Podisus maculiventris* egg masses were placed on the same leaves as the *H. halys* sentinel eggs to provide a direct choice for oviposition to parasitoids. In 2019, 285 *P. maculiventris* egg masses were deployed between 2 July and 3 September. No *P. maculiventris* eggs were deployed in 2020 or in 2021.

Yellow sticky traps were used for monitoring in 2020 and 2021. In 2020, eight traps were placed every 3 wk between 11 June and 4 September for a total of 384 at the western release sites. Similarly, eight traps were placed at each site biweekly, between June 13 and September 15 for a total of 400 across the 12 eastern release sites. At both the western and eastern sites half of the traps were placed at 1.5 m and half at 3.5 m height. The lower traps were attached to the same vegetation (tree or bush) as the sentinel eggs, and the higher traps were attached to the same bamboo pole that was used to raise sentinel eggs into tree canopies. In 2021, reduced monitoring was conducted by deploying eight traps at each of the 24 sites for 3-wk periods, 25 June–16 July at the western release sites and 23 June–13 July at the eastern release sites. On a few scheduled monitoring dates certain sites could not be accessed because of pesticide applications in the orchards and thus data are missing for sentinel eggs and yellow sticky traps.

### Estimating *Halyomorpha halys* Population Density

The relative population density of *H. halys* was estimated using pyramid traps baited with aggregation pheromones, with 12 sites monitored in 2019 (western only) and 24 sites monitored in 2020 (both western and eastern) (Supplementary Table S2). Each trap was baited with a dual stink bug lure (Trécé Pherocon Dual Lure). The cone at the top of the traps was lined on the inside with a piece of mesh infused with deltamethrin (D-Terrence net, Vestergaard S.A., Lausanne, Switzerland) to kill any stink bugs captured. At western sites the 4 traps per site were checked biweekly between 24 May and 9 September in 2019, and every 3 wk between 25 June and 7 October in 2020. At the eastern sites, traps were checked biweekly between 1 July and 9 October in 2020.

### Statistical Analyses

#### *Parasitism of* Halyomorpha halys *sentinel eggs.*

The rate of parasitism was assessed at the egg mass level by calculating the percentage of egg masses that yielded any parasitoids across the 12 eastern and 12 western sites. Parasitism rate on the individual egg level was calculated by using 28 eggs for the average size of an egg mass for *H. halys* (Nielsen et al. 2008). A generalized linear mixed model (GLMM) with binomial distribution (emergence vs. no emergence) and logit link function was used to assess differences in parasitism rates between frozen and fresh sentinel eggs and those placed either lower or higher in the canopy at the western sites in 2019. Height (low or high) and the state of eggs (fresh or frozen) were fixed variables and block (groups of 3 sites in proximity; see Supplementary Table S1 and Fig. 1) was a random variable.

GLMM s were used with a Poisson distribution and log link to assess how the state of the *H. halys* sentinel eggs, their placement, and sampling date (month) may influence the number and species identity of emerging parasitoids (both native and *Tr. japonicus*). Fixed effects in the model included date, placement, state of the eggs, and parasitoid species identity. Block was included as a random effect. All interactions were nonsignificant at α = 0.05 and did not improve AIC values, and therefore were not included in the final model. These analyses were done only for 2019 because in 2020 parasitoid captures were too few to analyze.

#### *Parasitism of* Podisus maculiventris *sentinel eggs.*

The rate of parasitism was assessed at the egg mass level by calculating the percentage of egg masses that yielded any parasitoids across the 4 western sites where 900 *Tr. japonicus* had been released and where *P. maculiventris* sentinel eggs had been deployed. Parasitism rate on the individual egg level was calculated by using 14 eggs for the average size of an egg mass (Legaspi 2004).

#### Parasitoids captured on yellow sticky traps.

GLMM s with binomial distribution and logit link function were used to compare parasitoid capture success (yes or no) of traps placed at different heights at the western and eastern release sites separately. The height and state of eggs were fixed variables and block was a random variable. The number of parasitoids captured by traps placed lower or higher was compared using linear mixed models. The fixed effect in the model was the placement (low vs. high) and block was included as a random variable.

#### Halyomorpha halys *density estimates.*

GLMM s with a Poisson distribution and log link were used with sampling date (2019 or 2020 categorical variable), block and their interactions as fixed effects to compare the number of *H. halys* captured in 2019 and 2020 at the western release sites. Since monitoring stopped in early September in 2019 but continued through September in 2020 only data for the months of June, July and August were included in the analysis. Block was included as a fixed effect to assess quantitatively regional differences in *H. halys* numbers. A similar analysis was conducted for stink bug captures at the eastern release sites for 2020 with block as a fixed factor. All analyses were performed using JMP Pro Version 17.0.0 (SAS Institute 2023).

## Results

### Parasitism of *Halyomorpha halys* Sentinel Eggs

In 2019 at the western sites, 840 *H. halys* sentinel egg masses were deployed and 727 were successfully retrieved. Parasitoids emerged from 30 egg masses from 9 of the 12 study sites (Table 1). This constitutes a 4.12% parasitism rate on the egg mass level over all sites, egg types, and placements. A total of 241 parasitoids emerged from the parasitized egg masses resulting in a 1.18% parasitism rate on the individual egg level. More than 1 parasitoid species emerged from 3 of the 30 egg masses parasitized in 2019. For those 3 observations the number of emerged parasitoids were divided equally between the 2 species and were included as such in the dataset.

**Fig. 2.**
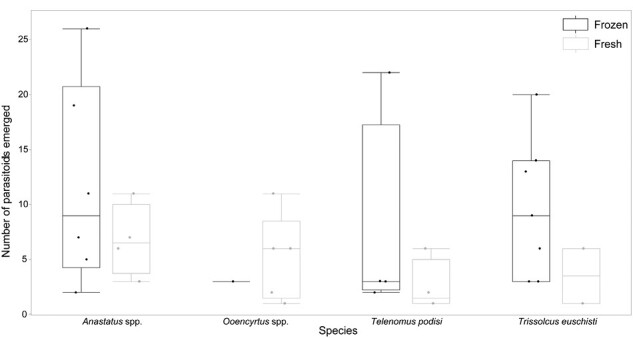
Number of parasitoids emerging from frozen or fresh *H. halys* sentinel eggs deployed in 2019 at the western sites. Dots indicate individual observations, the horizontal line indicates the median with the box representing the interquartile range, and vertical lines are 1.5 times the interquartile range.

At the western sites there was no significant difference in parasitism success of frozen versus fresh sentinel eggs (*F* = 3.5286, df = 1, 724, *P* = 0.0607). There was no difference in parasitism of eggs placed lower or higher in the canopy (*F* = 0.4713, df = 1, 720.7, *P* = 0.4926). The placement of eggs (*F* = 2.5962, df = 1, 25, *P* = 0.1197), the date (*F* = 0.7084, df = 2, 25, *P* = 0.0.5020) or the species identity of parasitoids (*F* = 1.3228, df = 3, 25, *P* = 0.2892) did not influence the number of emerging parasitoids. However, more parasitoids emerged from frozen eggs (*F* = 20.8293, df = 1, 25, *P* = 0.0001) (Table 1, Fig. 2).

**Fig. 3.**
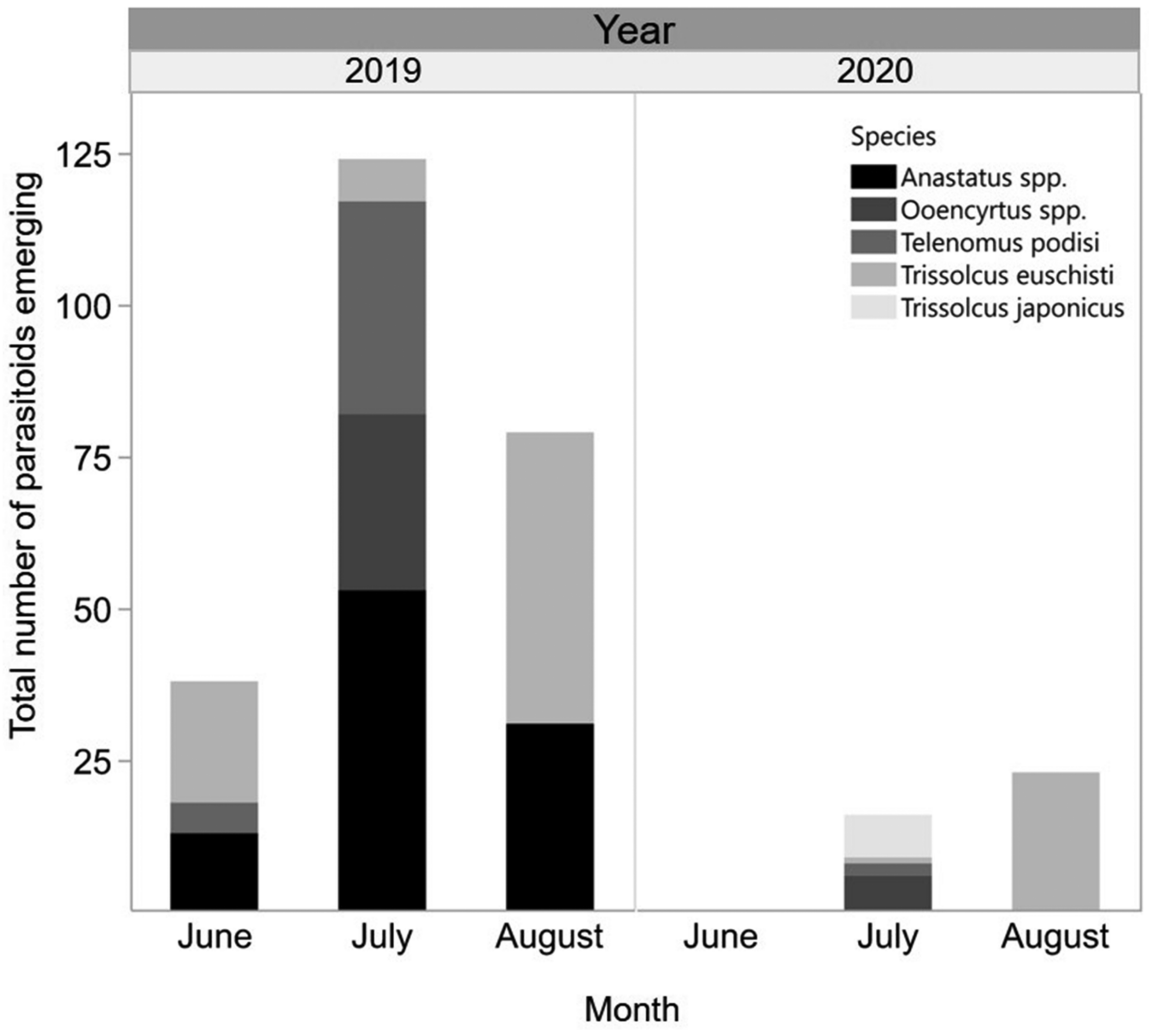
Total number of parasitoids of 5 species (or genera) emerging from *H. halys* sentinel eggs deployed in the same year of *Tr. japonicus* releases in 2 sets of release sites. Note that releases and sampling took place later in 2020 than in 2019 and only frozen eggs were used in 2020 for monitoring, which may explain the relatively lower parasitoid captures.

In 2019, all emerging parasitoids were native species representing 3 families and 4 genera: *Telenomus* (Scelionidae), *Trissolcus* (Scelionidae), *Anastatus* (Eupelmidae) and *Ooencyrtus* (Encyrtidae). The number of emerging native parasitoids were as follows: *Anastatus* spp. *n* = 97, *Tr. euschisti* (Ashmead) *n* = 55, *Te. podisi* Ashmead *n* = 40, and *Ooencyrtus* spp. *n* = 29 (Fig. 3).

In 2020, of the 430 *H. halys* sentinel egg masses deployed at the 12 eastern sites, 321 were retrieved. Six egg masses yielded a total of 39 parasitoids for a 1.87% parasitism rate at the egg mass level and 0.43% parasitism on the individual egg level across all sites (Table 1). *Trissolcus japonicus* emerged from 1 egg mass (*n* = 7 individuals) that was deployed on July 30 in a mixed orchard following releases of 500 parasitoid adults on July 1st (Fig. 1). *Trissolcus euschisti* emerged from 3 egg masses (*n* = 24), *Te. podisi* from 1 egg mass (*n* = 2) and Encyrtidae spp. from 1 egg mass (*n* = 6) (Fig. 3).

In 2021, of the 240 egg masses deployed over 24 sites, 219 were retrieved. The 30 egg masses that were parasitized yielded 394 parasitoids that constituted 13.7% parasitism on the egg mass level and 6.43% parasitism on the individual egg level (Table 1). *Trissolcus japonicus* emerged from 6 egg masses (*n* = 39) that were deployed at 2 sites. One of those sites received 100 *Tr. japonicus* in 2019 and the other 250 + 50 parasitoids in 2 consecutive releases in 2020 (Fig. 1). The number of native parasitoids emerging were as follows: *Anastatus* spp. *n* = 17, *Tr. euschisti n* = 208, *Tr. brochymenae n* = 27, *Te. podisi n* = 17, and Encyrtidae spp. *n* = 55.

### Parasitism of *Podisus maculiventris* Sentinel Eggs

Of the 285 *P. maculiventris* sentinel egg masses deployed in 2019, 133 were retrieved successfully. Three yielded 12 total parasitoids resulting in a 2.25% parasitism rate of egg masses across all sites. Parasitism rate on the individual egg level was 0.6% (Table 1). All parasitoids emerging from *P. maculiventris* eggs were native species from the genera *Telenomus* (*n* = 11) and *Ooencyrtus* (*n* = 1).

### Parasitoids Captured on Yellow Sticky Traps

**Fig. 4.**
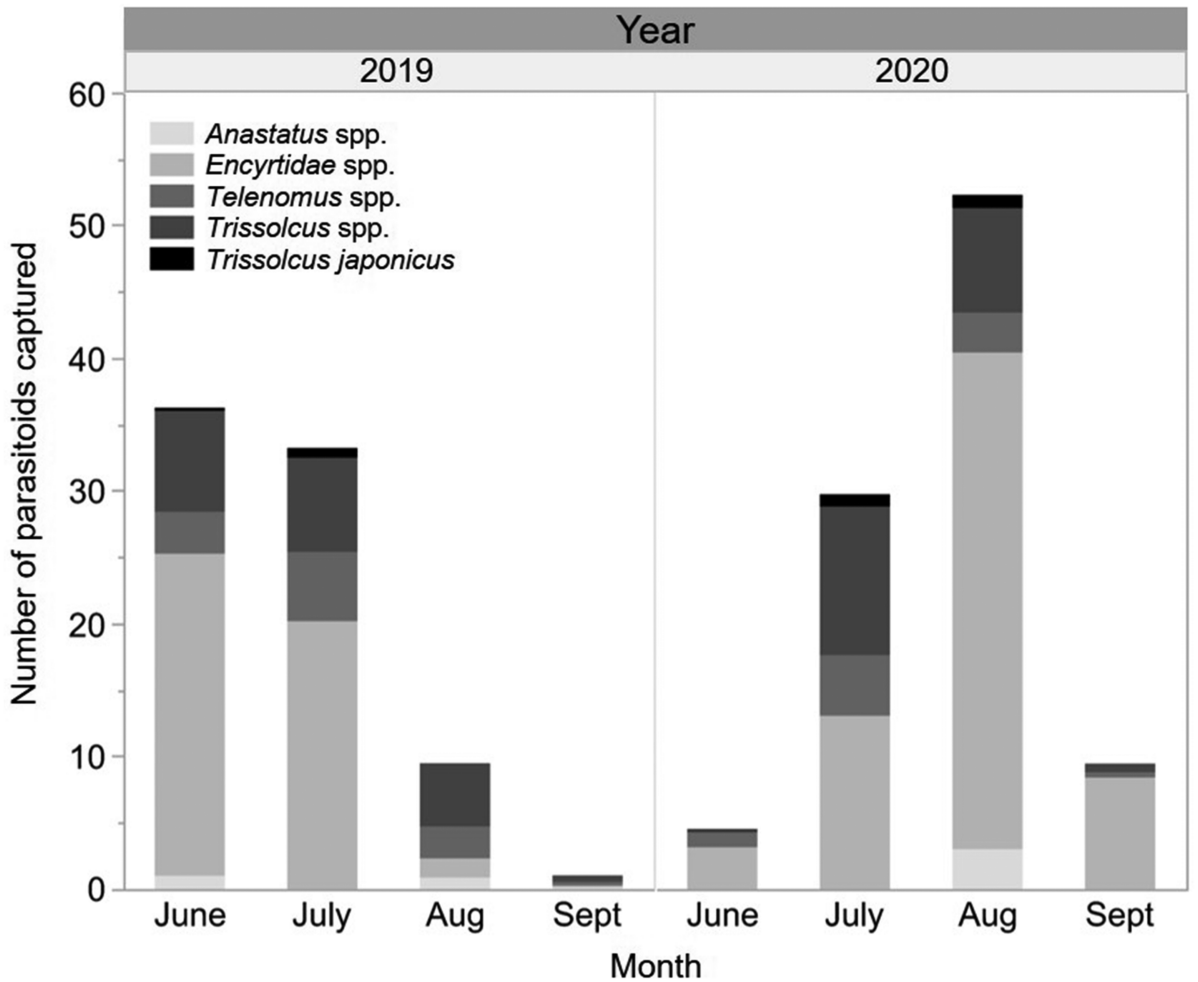
Parasitoid captures by yellow sticky traps in 2020 at 12 western study sites where *Tr. japonicus* was released in 2019 (left panel) and 12 eastern sites where released in 2020 (right panel).

Of the 384 yellow sticky traps placed at the 12 western field sites 1 yr following *Tr. japonicus* releases, 51 captured parasitoids that are likely to attack stink bugs. Encyrtidae spp. were captured most often (*n* = 46), followed by *Tr. euschisti* (*n* = 14), *Te. podisi* (*n* = 11), *Tr. thyantae* (Ashmead) (*n* = 2), *Tr. brochymenae* (Ashmead) (*n* = 2), *Anastatus* spp. (*n* = 2) and an unidentified *Trissolcus* spp. (*n* = 1) (Fig. 4). *Trissolcus japonicus* was captured at a single location where 900 individuals had been released in 2019 (Fig. 1). The number of parasitoids captured by traps placed lower or higher in the canopy did not differ (*F* = 0.8447, df = 1, 379.1, *P* = 0.3587).

Of the 400 yellow sticky traps placed at the 12 eastern study sites in the same year as *Tr. japonicus* releases took place, 69 captured parasitoids that likely attack stink bugs. The parasitoid species captured were similar to those captured at the western sites with Encyrtidae being most numerous (*n* = 62), followed by *Tr. euschisti* (*n* = 19), *Te. podisi* (*n* = 8), *Anastatus* spp. (*n* = 3), *Te. persimilis* Ashmead (*n* = 1) and *Tr. brochymenae* (*n* = 1) (Fig. 4). *Trissolcus japonicus* was recaptured at 2 sites. One of the sites had 250 and later 50 individuals released and the other was a control site located 4.3 km from the other capture site (Fig. 1). The number of parasitoids captured by traps placed lower or higher in the canopy did not differ (*F* = 0.0176, df = 1, 395, *P* = 0.8945).

### *Halyomorpha halys* Density Estimates

**Fig. 5.**
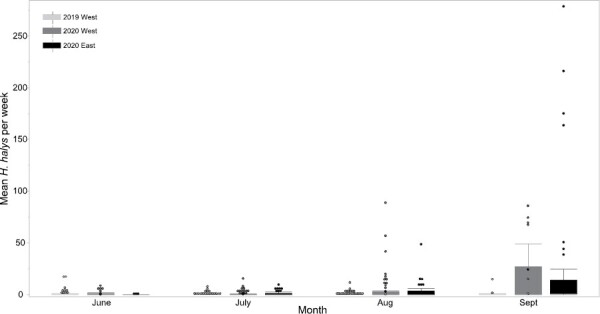
Mean number of *H. halys* captured weekly by pyramid traps at sites where *Tr. japonicus* was released either in 2019 (western sites) or 2020 (eastern sites). Western release sites were sampled in 2019 and 2020 (2019 West and 2020 West). Eastern release sites were sampled in 2020 (2020 East). For total *H. halys* at each study site see Supplementary Table S1. Dots indicate outliers, the horizontal line indicates the median with the box representing the interquartile range, and vertical lines are 1.5 times the interquartile range.

*Halyomorpha halys* densities across the 12 western sites were higher in 2020 (nonmodel means per trap per week for the season: 4.71 ± 13.7 SE) than in 2019 (0.54 ± 1.92) (year: χ^2^ = 209.8202, df = 1, *P* < 0.0001). There were also significant regional differences in *H. halys* densities (block: χ^2^ = 123.1357, df = 3, *P* < 0.001) (Supplementary Table S2, Fig. 5). Mean *H. halys* captures at the western sites were significantly lower in block 2 (see Supplementary Table S1 and Fig. 1) (0.21 ± 1.9 SE) than the other 3 blocks (block 1: 3.2 ± 8.9, block 3: 3.2 ± 12.62, block 4: 2.98 ± 10.48) (Fig. 3). *Halyomorpha halys* captures were higher at 3 of the 4 blocks in 2020 than in 2019, but in block 2 they remained low (block x year interaction: χ^2^ = 59.3004, df = 3, *P* < 0.0001). Total *H. halys* captures at the western sites were between 1 and 168 individuals in 2019 and between 2 and 817 in 2020 (Supplementary Table S2). The higher *H. halys* captures in 2020 are partly due to the longer sampling period but *H. halys* numbers were higher across the season in 2020 than in 2019 (Fig. 5).

There was also regional variation in *H. halys* densities at the eastern release sites (block: χ^2^ = 1770.6597, df = 3, *P* < 0.0001). Block 5 (see Supplementary ,Tables S1, S2 and Fig. 1) had significantly higher mean *H. halys* densities (nonmodel means per trap per week: 25.4 ± 55.4) than the other blocks (block 6: 4.61 ± 10.3, block 7: 5.4 ± 14, block 8: 3.8 ± 12.3) (Supplementary Table S1, Fig 1). Total *H. halys* captures at eastern release sites ranged between 21 and 2830 in 2020 (Supplementary Table S2).

## Discussion

We conducted experimental releases of *Tr. japonicus* to increase its density and distribution across lower Michigan to accelerate biological control of *H. halys*. One of our goals with these experimental releases was to test the role of release size and release frequency for establishment success. Despite releasing 7,200 adult parasitoids over a 2-yr period (2019–2020) at 24 field sites and 3 yr of monitoring between 2019 and 2021 using 1,510 *H. halys* sentinel egg masses and 784 yellow sticky traps, *Tr. japonicus* was captured at only 5 of the sites on 7 sentinel egg masses and 3 yellow sticky traps (49 adults total) (Fig. 1). At 3 of the sites the captures occurred 1–2 yr following release suggesting overwintering success. At 2 of the sites *Tr. japonicus* was captured within the same year of the releases which signals reproductive activity. Given the low capture rates we could not assess the best release approach that increases establishment success.

The release sizes used in 2019 and 2020 that ranged from 100 to 900 individuals (Table 1, Fig. 1) are comparable to other studies testing the importance of release size of parasitoids for establishment. For example, 1, 10, or 100 female *Neodryinus typhlocybae* were introduced against a planthopper in southern France (Fauvergue et al. 2007) and 100–1000 *Torymus sinensis* were released at each site against the chestnut gall wasp (*Dryocosmus kuriphilus*) (Borowiec et al. 2018). Both the above studies with *N. typhlocybae* and *To. sinensis* found parasitoid establishment and increasing population sizes in the same year and 1 or 2 yr after the original releases (Fauvergue et al. 2007, Borowiec et al. 2018). However, our results show that very limited distribution and density of *Tr. japonicus* a few years after initial detection are not unusual. For example, a 2017 study in New Jersey that placed 236 sentinel eggs in commercial peach and apple orchards found only 3 egg masses parasitized by *Tr. japonicus* (Kaser et al. 2018). In Virginia, only 3 of 135 sentinel egg masses were parasitized by *Tr. japonicus* in 2016 (Quinn et al. 2019). These studies, conducted within 2–3 yr of the initial detection of *Tr. japonicus* in nearby Maryland in 2014 (Talamas et al. 2015), indicate very low densities within a few years after discovery.

*Trissolcus japonicus* establishment and population growth in Michigan may have been impacted by the low density of *H. halys* at the release sites (Fig. 4). Spring 2019 was unusually cold and wet and may have increased mortality or delayed development of *H. halys*, which might partly explain the lack of *Tr. japonicus* captures that year. While in 2020 *H. halys* densities were several times higher than in 2019, overall stink bug densities were highly variable among release sites with only 1 site exceeding 1,000 *H. halys* captures throughout the season (Supplementary Table S2). The relatively low stink bug densities combined with the single generation of *H. halys* produced in Michigan (Nielsen and Hamilton 2009, Nielsen et al. 2016) likely limits the speed of *Tr. japonicus* population growth. In addition, the low founding size of our *Tr. japonicus* colony (Jarrett et al. 2019) likely resulted in lower genetic diversity that could have reduced fitness of parasitoids (Fauvergue et al. 2012) compared to other releases that were able to use individuals from more diverse colonies. Alternatively, it is possible that the released *Tr. japonicus* individuals dispersed from the release sites to locations that our sampling did not cover.

The native parasitoid community that attacked *H. halys* sentinel eggs in Michigan is represented by 4 genera (*Trissolcus*, *Anastatus*, *Telenomus*, and *Ooencyrtus*) and is similar to those found across North America (Abram et al. 2017). Average rates of parasitism by native parasitoids in Michigan that were between 1.87 and 4.12% at the egg mass level and 0.43–1.18% at the individual egg level when surveyed through the season (Table 1) also align well with results of a review that found parasitism levels <5% in 87% of surveys (Abram et al. 2017). However, it should be noted that sentinel eggs tend to underestimate parasitism compared to field-laid wild egg masses of *H. halys* (Jones et al. 2014). Our use of laboratory-laid sentinel eggs likely influenced parasitism rates both by native parasitoids and by *Tr. japonicus*. In our study, more parasitoids emerged from frozen than from fresh *H. halys* eggs, but the likelihood of parasitism did not differ between frozen and fresh eggs (Fig. 2). In Maryland, surveys also found higher emergence from frozen eggs, which may be explained by the lack of adaptation by native parasitoids to the immune response presented by viable *H. halys* eggs (Herlihy et al. 2016). However, a review of 98 datasets did not find any difference in parasitism rates between fresh and frozen sentinel eggs (Abram et al. 2017, McIntosh et al. 2019).

Previous studies indicated that *Tr. japonicus* captures are most likely to occur in the middle (at 4.8 m height) and upper canopy based on sampling of 5–10 trees-of-heaven, while native parasitoids may be captured at any location (low, mid, or upper canopy) (Quinn et al. 2019, Dyer et al. 2022). We placed sentinel eggs on 240 individual trees, and yellow sticky traps on 192 trees. Although tree-of-heaven grows in Michigan, we did not specifically target that species in our sampling efforts, instead relying on various deciduous tree species common to the wood lots adjacent to the orchards that were the focal point of the study. Our results corroborate that native parasitoids are equally likely to be captured both in the low and mid-canopy, though our mid-canopy location was lower at 3.5 m height than in Quinn et al. (2019). Four of our five *Tr. japonicus* recaptures occurred at the 1.5 m height, which would be considered a low canopy sample by Quinn et al. (2019) and contradicts their recommendation to sample only in the mid and high canopy for *Tr. japonicus*. These discrepancies indicate that it may be difficult to generalize patterns with regards to whether fresh or frozen eggs are more likely to be attacked, and at which heights parasitism by native or exotic parasitoids may be more prevalent. Differences in climate, site characteristics, sampling methods, parasitoid and host densities, and a multitude of other factors between studies may account for the different findings. However, there is general agreement across studies that the current rates of egg parasitism by native parasitoids are unlikely to provide sufficient biological control to suppress *H. halys* populations (Jones et al. 2014, Cornelius et al. 2016, Ogburn et al. 2016, Abram et al. 2017, Dieckhoff et al. 2017).

While *Tr. japonicus* is considered the most promising biocontrol agent because of its high parasitism rates of 50–80% of *H. halys* eggs in the native range (Yang et al. 2009, Zhang et al. 2017), there are concerns regarding potential nontarget effects given its oligophagous host range that includes several species native to North America (Hedstrom et al. 2017, Botch and Delfosse 2018). We tested for nontarget effects on one of the most suitable native species, *Podisus maculiventris*, a beneficial predatory stink bug that was shown to support up to 64% development success of *Tr. japonicus* in the laboratory (Linder et al. 2023). Surveys also found parasitism of *P. maculiventris* by *Tr. japonicus* in the field (Gariepy and Talamas 2019, Milnes and Beers 2019). We deployed over 100 *P. maculiventris* eggs at 4 sites where 900 *Tr. japonicus* had been released in 2019 but only native parasitoids attacked those eggs. In a survey in 2018, when 51 *P. maculiventris* eggs were placed alongside *H. halys* sentinel eggs we also did not find parasitism by *Tr. japonicus* (Jarrett et al. 2019). However, given the low density of *Tr. japonicus* in Michigan, these results may not be representative of its potential impact on native species and it should be reevaluated once population densities increase. Because parasitism of native stink bugs appears much lower (0.4–8%) in the field than that of *H. halys* (77%), and because several studies demonstrated strong preference towards and higher fitness of *Tr. japonicus* on *H. halys* compared to native stink bugs (Boyle et al. 2020, Malek et al. 2021, Linder et al. 2023) its redistribution continues within multiple states (Jentsch 2017, Milnes and Beers 2019). *Trissolcus japonicus* is expected to be an important component of integrated pest management programs targeting *H. halys* both in North America and in Europe (Abram et al. 2020) and the field releases conducted as part of this study will likely contribute to its widespread establishment and population growth across Michigan.

## Supplementary Material

nvad102_suppl_Supplementary_Material

## Data Availability

Data are available upon request.
