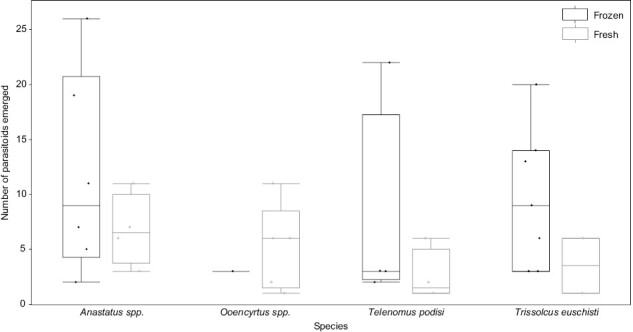# Correction to: Field releases of the exotic parasitoid *Trissolcus japonicus* (Hymenoptera: Scelionidae) and survey of native parasitoids attacking *Halyomorpha halys* (Hemiptera: Pentatomidae) in Michigan

**DOI:** 10.1093/ee/nvad118

**Published:** 2023-12-09

This is a correction to: Olivia Simaz, Julie Michaelson, Julianna K Wilson, Elijah Talamas, Larry Gut, John Pote, Marianna Szűcs, Field releases of the exotic parasitoid *Trissolcus japonicus* (Hymenoptera: Scelionidae) and survey of native parasitoids attacking *Halyomorpha halys* (Hemiptera: Pentatomidae) in Michigan, *Environmental Entomology*, 2023, nvad102, https://doi.org/10.1093/ee/nvad102

In the originally published version of this manuscript, Figure 2 was incorrectly included from an unrelated article. The correct Figure 2 for this article is as follows:

This error has been corrected online.