# Supplementary material for: Field releases of the exotic parasitoid Trissolcus japonicus (Hymenoptera: Scelionidae) and survey of native parasitoids attacking Halyomorpha halys (Hemiptera: Pentatomidae) in Michigan
Source: Environ Entomol. 2023 Oct 6;52(6):998–1007. doi: 10.1093/ee/nvad102 (PMC10724022; doi:10.1093/ee/nvad102)
Supplement: nvad102_suppl_Supplementary_Material [file nvad102_suppl_supplementary_material.docx]

**Supplementary Materials**

**Table. S1.** Study sites, experimental design and site characteristics. All sites designated as ‘Mixed’ had both a diversity of fruits and vegetables grown. Sites characterized as ‘Low input’ use a mix of conventional and organic management methods.

| **Study site** | **Block** | **Release size** | **Release date** | **Latitude** | **Longitude** | **Crop** | **Management** |
| --- | --- | --- | --- | --- | --- | --- | --- |
| Bath | 1 | 0 | 2019 | 42.8566 | -84.4083 | Mixed | Low input |
| Charlotte | 1 | 100 | 2019 | 42.6337 | -84.7877 | Mixed | Low input |
| St. Johns | 1 | 900 | 2019 | 43.1207 | -84.6252 | Apple | Conventional |
| Conklin | 2 | 0 | 2019 | 43.1526 | -85.8195 | Apple | Conventional |
| Grand Rapids | 2 | 100 | 2019 | 43.0629 | -85.7396 | Apple | Conventional |
| Sparta | 2 | 900 | 2019 | 43.1113 | -85.7580 | Apple | Conventional |
| Grand Junction | 3 | 0 | 2019 | 42.3450 | -86.0502 | Apple | Conventional |
| Covert | 3 | 100 | 2019 | 42.3066 | -86.2537 | Apple | Conventional |
| Bangor | 3 | 900 | 2019 | 42.2949 | -86.1816 | Apple | Conventional |
| Benton Harbor | 4 | 0 | 2019 | 42.0829 | -86.3521 | Mixed | Conventional |
| Coloma | 4 | 100 | 2019 | 42.1615 | -86.3123 | Mixed | Low input |
| Hartford | 4 | 900 | 2019 | 42.1852 | -86.1749 | Apple | Conventional |
| Burton | 5 | 0 | 2020 | 43.0256 | -83.5747 | Apple | Low input |
| Davison | 5 | 250+50 | 2020 | 42.9692 | -83.4894 | Apple | Low input |
| Flint | 5 | 500 | 2020 | 43.0321 | -83.6785 | Apple | Low input |
| Washington 1 | 6 | 0 | 2020 | 42.8003 | -83.0698 | Apple | Conventional |
| Washington 2 | 6 | 250+50 | 2020 | 42.7866 | -83.0215 | Apple | Conventional |
| Armada | 6 | 500 | 2020 | 42.8608 | -82.9493 | Apple | Conventional |
| Britton | 7 | 0 | 2020 | 42.1425 | -83.6286 | Mixed | Conventional |
| Milan | 7 | 250+50 | 2020 | 41.9598 | -83.9226 | Mixed | Conventional |
| Chelsea | 7 | 500 | 2020 | 42.2579 | -84.1103 | Mixed | Organic |
| Linden | 8 | 0 | 2020 | 42.6898 | -83.8726 | Apple | Abandoned |
| Fenton | 8 | 250+50 | 2020 | 42.6919 | -83.7498 | Apple | Conventional |
| Howell | 8 | 500 | 2020 | 42.8059 | -83.7945 | Apple | Conventional |

**Table S2.** Sampling dates and total *H. halys* captured for each study site in 2019 and 2020. Sites within the same experimental block are shaded grey.

| **Study site** | **Block** | **Sampling dates in 2019** | **Total *H. halys* captured in 2019** | **Sampling dates in 2020** | **Total *H. halys* captured in 2020** |
| --- | --- | --- | --- | --- | --- |
| Bath | 1 | May 24 - Sept 9 | 168 | June 25 - Oct 7 | 699 |
| Charlotte | 1 | May 24 - Sept 9 | 19 | June 25 - Oct 7 | 267 |
| St. Johns | 1 | May 24 - Sept 9 | 3 | June 25 - Oct 7 | 16 |
| Conklin | 2 | May 24 - Sept 9 | 1 | June 25 - Oct 7 | 72 |
| Grand Rapids | 2 | May 24 - Sept 9 | 2 | June 25 - Oct 7 | 7 |
| Sparta | 2 | May 24 - Sept 9 | 4 | June 25 - Oct 7 | 2 |
| Grand Junction | 3 | May 24 - Sept 9 | 8 | June 25 - Oct 7 | 96 |
| Covert | 3 | May 24 - Sept 9 | 21 | June 25 - Oct 7 | 392 |
| Bangor | 3 | May 24 - Sept 9 | 12 | June 25 - Oct 7 | 813 |
| Benton Harbor | 4 | May 24 - Sept 9 | 30 | June 25 - Oct 7 | 171 |
| Coloma | 4 | May 24 - Sept 9 | 67 | June 25 - Oct 7 | 817 |
| Hartford | 4 | May 24 - Sept 9 | 2 | June 25 - Oct 7 | 42 |
| Burton | 5 | NA |  | July 1 - Oct 9 | 2830 |
| Davison | 5 | NA |  | July 1 - Oct 9 | 420 |
| Flint | 5 | NA |  | July 1 - Oct 9 | 204 |
| Washington 1 | 6 | NA |  | July 1 - Oct 9 | 21 |
| Washington 2 | 6 | NA |  | July 1 - Oct 9 | 469 |
| Armada | 6 | NA |  | July 1 - Oct 9 | 135 |
| Britton | 7 | NA |  | July 1 - Oct 9 | 305 |
| Milan | 7 | NA |  | July 1 - Oct 9 | 410 |
| Chelsea | 7 | NA |  | July 1 - Oct 9 | 22 |
| Linden | 8 | NA |  | July 1 - Oct 9 | 472 |
| Fenton | 8 | NA |  | July 1 - Oct 9 | 24 |
| Howell | 8 | NA |  | July 1 - Oct 9 | 21 |
